# Supplementary material for: Intersectoral Cost of Treating Pulmonary Non-Tuberculosis Mycobacterial Disease (NTM-PD) in Germany—A Change of Perspective in Disease Management
Source: Int J Environ Res Public Health. 2019 Oct 9;16(20):3795. doi: 10.3390/ijerph16203795 (PMC6843529; doi:10.3390/ijerph16203795)
Supplement: Supplementary file 1 [file ijerph-16-03795-s001.pdf]

Supplementary Material – Additional Explanations

Calculation methods

This analysis considers only the charging of costs which are legally mandated or that are due to procedures representing "state of the art" of diagnostics and therapy of active disease. The following cost categories are defined and calculated based on the most recent rates established by the new Statutory Health Insurance (SHI) Scheme (Einheitlicher Bewertungsmaßstab, EBM) [1] for outpatient services which came into effect on 01 July 2019. This scheme constitutes the costs effectively incurred by the SHI, meaning that the compensation for physician services addresses "prices" rather than the actual incurred costs due to resource consumption.
Differentiating NTM lung disease from pulmonary tuberculosis remains difficult, because the clinical presentations of the two diseases are similar and a definite diagnosis of NTM lung disease based on sputum cultures or cultures gained by bronchoalveolar lavage takes time. Therefore, the algorithm of diagnostic and monitoring procedures for NTM-PD used in the cost analysis is derived from both the recommendations for diagnosis and treatment of nontuberculous mycobacterioses of the German Central Committee against Tuberculosis [2] and the current guidelines of the German Central Committee against Tuberculosis (DZK) for TB therapy [3].

Treatment options and their weight in Monte-Carlo simulation

MAC isolates are usually macrolide susceptible in patients receiving treatment for the first time. Therefore, for initial treatment of patients with MAC-PD a three-drug regimen containing a macrolide (clarithromycin [CLAM] or azithromycin [AZT]), ethambutol [EMB] and a rifampin (rifampicin [R] or rifabutin [RBT]) is generally recommended. The German guidelines [2] and those of the British Thoracic Society (BTS) [4] call for a daily regimen of rifampicin 600mg (or rifabutin 300 mg if the patient is taking drugs that interact with rifampicin or has had hepatotoxicity due to rifampicin, respectively) plus EMB 15 mg/kg and AZT 250 mg or CLAM 1000mg). An injectable amikacin should be considered in patients with severe MAC pulmonary disease (e.g., if there is radiological evidence of lung cavitation) or in macrolide-resistant MAC disease at least for the first two months [5]. Antibiotic treatment for MAC disease should be continued for a minimum of 12 months after culture conversion and sputum samples be sent for gaining cultures every 4 weeks during treatment up to 12 months after completing treatment to assess the microbiological response. Since sputum conversion generally requires 2-to 6 months of treatment, MAC patients will ultimately be treated for 14 to 18 months. In macrolide-resistant MAC disease, beside amikacin, treatment with R, E and a fluoroquinolone, e.g. moxifloxacin (MOX) (BTS) or clofazimine (CLO [6]) are recommended.

Therefore, in our calculations, four different drug combinations of a standard treatment have to be taken into consideration including AZT instead of CLAM and RBT instead of R, each for 14 or 18 month-treatment, representing 84% of all MAC-PD cases. The weight of each combination can therefore be calculated as 0.84/8, or 0.105%. 16% of patients, however, are estimated to be either severe or macrolide-resistant and thus require additional i.v. amikacin for at least 2 months. In macrolide-resistant cases MOX or CLZ can be used, resulting in 2 combinations each for 14 or 18 months. The other combinations are the drug variations of the standard regimen as stated above. Thus in our model, each of the total of 12 options has a weight of 1/12 for the remaining 16% (0.0133).

Diagnostic procedures

Patients with suspected pulmonary TB or NTM are generally given a chest X-ray (affected organ) followed by bacteriological confirmation - usually from sputum or bronchoalveolar lavage following referral to a pneumologist - with microscopic verification of acid-fast bacilli (almost always an indicator of the presence of mycobacteria) and/or culture (pathogen verification) together with a susceptibility test of the first isolate for standard drugs against NTM (mostly against bacteria of the M. avium complex). In any case of suspected TB or NTM, nucleic acid amplification tests (NAAT) should, in addition, be performed routinely for rapid identification of the tuberculosis complex, e.g. by XPERT® MTB/RIF Ultra (Cepheid). The XPERT has the advantage that its result remains negative if a patient with a positive sputum or BAL smears suffers from NTM. Therefore, in those cases the XPERT can be used for purpose of differential diagnosis.
As the costs of treatment are addressed here, the cost calculation of monitoring refers to potential side effects that can occur throughout therapy by those drugs.

References

1. Federal Association of Statutory Health Insurance Physicians. Physicians Fee Schedule. Available at: http://k bv.de/html/ebm.php. Accessed July 17, 2019.
2. Schönfeld N, Haas W, Richter E, et al. [Recommendations for diagnosis and treatment of nontuberculous mycobacterioses of the German Central Committee against tuberculosis and the German Respiratory Society]. *Pneumologie*. 2013 Nov;67:605-33.
3. Schaberg T, Bauer T, Brinkmann F, Diel R, et al. [Tuberculosis Guideline for Adults - Guideline for Diagnosis and Treatment of Tuberculosis including LTBI Testing and Treatment of the German Central Committee (DZK) and the German Respiratory Society (DGP)]. *Pneumologie*. 2017;71:325-397.
4. Haworth CS, Banks J, Capstick T, et al. British Thoracic Society Guideline for the management of non-tuberculous mycobacterial pulmonary disease (NTM-PD). *BMJ Open Resp Res*. 2017;4:e000242.
5. Ryu YJ, Koh WJ, Daley CL. Diagnosis and Treatment of Nontuberculous Mycobacterial Lung Disease: Clinicians' Perspectives. *Tuberc Respir Dis* (Seoul). 2016;79:74-84.
6. Kasperbauer S, Daley C. Treatment of Mycobacterium avium complex lung infection in adults. UpToDate. Available at: https://www.uptodate.com/contents/treatment-of-mycobacterium-avium-complex-lung-infection-in-adults#! Accessed 4 August 2, 2019.
